# Effect of light regime and provenance on leaf characteristics, growth and flavonoid accumulation in *Cyclocarya paliurus* (Batal) Iljinskaja coppices

**DOI:** 10.1186/s40529-016-0145-7

**Published:** 2016-10-17

**Authors:** Yang Liu, Chenyun Qian, Sihui Ding, Xulan Shang, Wanxia Yang, Shengzuo Fang

**Affiliations:** 1 College of Forestry, Nanjing Forestry University, Nanjing, 210037 People’s Republic of China; 2 Co-Innovation Center for Sustainable Forestry in Southern China, Nanjing Forestry University, Nanjing, 210037 People’s Republic of China

**Keywords:** *Cyclocarya paliurus*, Chlorophyll content, Environmental factor, Flavonoid content, Genotype, Leaf biomass, Palisade cell

## Abstract

**Background:**

As a highly valued and multiple function tree species, *Cyclocarya paliurus* is planted and managed for timber production and medical use. However, limited information is available on its genotype selection and cultivation for growth and phytochemicals. Responses of growth and secondary metabolites to light regimes and genotypes are useful information to determine suitable habitat conditions for the cultivation of medicinal plants.

**Results:**

Both light regime and provenance significantly affected the leaf characteristics, leaf flavonoid contents, biomass production and flavonoid accumulation per plant. Leaf thickness, length of palisade cells and chlorophyll a/b decreased significantly under shading conditions, while leaf areas and total chlorophyll content increased obviously. In the full light condition, leaf flavonoid contents showed a bimodal temporal variation pattern with the maximum observed in August and the second peak in October, while shading treatment not only reduced the leaf content of flavonoids but also delayed the peak appearing of the flavonoid contents in the leaves of *C. paliurus*. Strong correlations were found between leaf thickness, palisade length, monthly light intensity and measured flavonoid contents in the leaves of *C. paliurus.* Muchuan provenance with full light achieved the highest leaf biomass and flavonoid accumulation per plant.

**Conclusions:**

*Cyclocarya paliurus* genotypes show diverse responses to different light regimes in leaf characteristics, biomass production and flavonoid accumulation, highlighting the opportunity for extensive selection in the leaf flavonoid production.

## Background

*Cyclocarya paliurus* (Batal) Iljinskaja, commonly called “sweet tea tree” because of the flavor of its leaves, is a sole species from *Cyclocarya* genus and is widely distributed in sub-tropical regions of China (Fang et al. 2006). Leaves of this plant are traditionally used in China as a medicine or nutraceutical tea (Birari and Bhutani 2007; Fang et al. 2011). Many studies have demonstrated that *C. paliurus* possesses a variety of bioactivities, including antihypertensive activity, hypoglycemic activity, enhancement of mental efficiency, anticancer, anti-HIV-1, and antioxidant activity (Kurihara et al. 2003; Xie et al. 2010, 2013; Zhang et al. 2010). These beneficial effects have been partly attributed to its content of several chemical components, including proteins, polysaccharides, triterpenoids, flavonoids, steroids, saponins, phenolic compounds, and minerals (Xie et al. 2010, 2013; Li et al. 2011; Fang et al. 2011). Owing to its multiple beneficial effects on human health, a huge production of leaves is required for *C. paliurus* tea production and for medical use. However, most studies on *C. paliurus* were focused on extraction procedures and low molecular weight substances, such as triterpenoids, flavonoids, steroids, saponins, and other compounds present in this plant, whereas less attention was paid to the silvics of the species (Deng et al. 2012, 2015).

Flavonoids are a large group of phenolic constituents of plants, and the bioavailability of flavonoids varies greatly among different subgroups and compounds (Erlund 2004). Some beneficial bioactivities of flavonoids have been proved, such as antibacterial, anticarcinogenic, antioxidant, antimutagenic, anti-inflammatory, antiallergic, antiobesity, and antidiabetic activities (Peterson and Dwyer 1998; Erlund 2004; Zhang et al. 2010). Quercetin and its glycoside derivatives, such as isoquercitrin, have been found to promote human health through cytoprotective effects, including reducing lipid peroxidation, protein carbonylation and radical oxygen species (ROS) production (Ciancolini et al. 2013; Palazzolo et al. 2012), while kaempferol was found to have remarkable antioxidant potential and has the capacity to lower the risks of coronary heart disease (Schmidt et al. 2010). According to Iwashina (2000), biosynthesis of flavonoids is mostly carried out by plant, with a few exceptions of animals and fungi.

The content of phytochemicals in plants is affected by numerous internal and external factors that occur during the growing period (Graham 1998; Björkman et al. 2011; Cui et al. 2013; Liu et al. 2015). Thus, factors influencing the phytochemical content and profile in the production of plants are worth considering for improved cultivation. Recently, attempts have been made to develop plantations of *C. paliurus* as a functional food or ingredient to be used in traditional Chinese medicine. Fang et al. (2011) investigated the genetic and temporal variations in the flavonoid (quercetin, kaempferol, and isoquercitrin) content in leaves of *C. paliurus*, while Deng et al. (2012) explored the effects of environment and fertilization on growth and flavonoid content of *C. paliurus*. Xie et al. (2015) had examined the effects of plant growth regulating substances on growth and phytochemical content of *C. paliurus* leaves. Björkman et al. (2011) indicated that factors that influence plant growth and phytochemical content may interact, and it is now possible to design multifactorial experiments that simulate their combined effects. However, very little is known about the influences of interaction between genetic and environmental factors on plant growth and phytochemical contents of *C. paliurus* leaves, especially the mechanism of flavonoid accumulation in *C. paliurus* plants.

For these reasons, it is necessary to investigate the integrated influences of provenance and light intensity on plant growth, leaf characteristics, accumulation of total flavonoid and key health promoting flavonoids (quercetin, kaempferol, and isoquercitrin) in the leaves of *C. paliurus*. The information provided by this study would be of great value for understanding the light-regulating mechanism for different provenances of *C. paliurus*, and contribute to establishing optimal cropping strategies for *C. paliurus* plants.

## Methods

### Plant material and growth conditions

The experiment was carried out during the 2011 growing season in Zhenjiang Nursery, Jiangsu Province, China, and the site conditions were the same as described by Fang et al. (2011). For each provenance, seed trees (generally dominant or co-dominant tree in the stand) were selected based on tree age, stem form and growth vigor. Number of trees for collecting seeds for each provenance was determined according to stand area and quantity of *C. paliurus* naturally distributed on the area (about 10 % of the total). Seeds of *C. paliurus* were collected in late October 2009 and were subjected to chemical scarification, exogenous gibberellin A3 (GA3) treatments, and stratification treatments in early January 2010, according to the method proposed by Fang et al. (2006). After a 3 month stratification treatment, the germinated seeds were sown in containers, and then transplanted into the experimental site at a spacing of 40 × 50 cm in early June 2010. After 1-year growth, the seedlings of *C. paliurus* were cut from the bottom in March 2011 for coppice management.

A split-plot randomized design was used to establish nine treatments with three shading levels and three provenances. Three shading treatments were subjected to three light intensity regimes: 100 % sunlight (A1, without shading net), about 50 % of solar radiation (A2, covered with one layer of shading net at 2 m height), and around 15 % of solar radiation (A3, covered with two layers of shading net at 2 m height). Three provenances were Wufeng (30° 19′ N, 110° 89′ E) from Hubei Province (B1), Yuanling (28° 46′ N, 110° 39′ E) from Hunan Province (B2) and Muchuan (28° 96′ N, 103° 78′ E) from Sichuan Province (B3), respectively. With three replications, the trial gave a total of 27 subplots, and each subplot consisted of 20 plants.

Shading treatments were conducted from June 2011. To monitor environmental factors automatically, a hand-held Agricultural Weather Station (TNHY series model, Zhejiang Top Instrument Co. Ltd., Hangzhou, China) was set up in different shading treatments. Photosynthetic photon flux density (PPFD) was recorded at full sunlight and under shade conditions at intervals of 30 min, whereas air temperature (T) and relative humidity (RH) were measured at intervals of 10 min during the experimental periods by the set hand-held Agricultural Weather Station.

### Growth and biomass assessment

Growth and biomass assessments of the plants were conducted on October 20, 2011. Based on the measurements of base diameter and height, three intact *C. paliurus* plants with most close to mean base diameter and height in each treatment were selected and harvested for biomass analysis. After excavating, the sample plants were washed with tap water. Then leaf, stem, and root components of each sample tree were separated, weighed, and dried at 70 °C. The total dry mass of each was calculated as the sum of leaf, stem, and root dry weights.

### Measurement of leaf characteristics and chlorophyll concentration

Leaf area (LA) per plant (cm^2^) of fully expanded leaves was measured by an area meter (Li-Cor, Model 3100 area meter, USA). Leaves of similar size were used for anatomical measurements. Cross sections were cut into about 4 mm × 6 mm by hand from the same levels of leaves (1 cm below the leaf tip) in all materials. The sections were fixed in a FAA (formalin/glacial acetic/70 % ethanol in the ratio of 0.5:0.5:9.0) and dehydrated through the gradual ethanol series and *t*-butanol. Samples were then sectioned with a microtome (Leica CM1850, Leica Instruments, Nussloch, Germany) into slices of 30 μm thickness. The section tissues were stained 10 min with a 1 % aluminium chloride-ethanol solution. Microphotographs were taken with a Nikon YS100 microscope (Nikon Co. Tokyo, Jena, Japan), while leaf thickness and palisade length were examined using a FW4000 software.

For the analysis of chlorophyll concentrations, 0.1 g of finely cut and well-mixed sample was extracted with 8 mL 95 % acetone for 24 h at 4 °C in darkness and shaken 3 or 4 times until they were bleached, according to the method of Tang et al. (2015). The absorbance was measured with a spectrophotometer Shimadzu UV-2550 (Kyoto, Japan) at 646.6, 663.6 and 450 nm respectively, after centrifugation of the mixture on standing. Chlorophyll concentrations were calculated by the standard method of Porra et al. (1989) and expressed in mg g^−1^ fresh weight (FW).

### Determination of flavonoid concentration

Approximately 50 g of fresh fully developed leaves of each provenance and shading treatment was sampled at about 1 month intervals (20 July, 20 August, 20 September, 20 October and 15 November in 2011). All samples were dried, sliced and ground into fine powder before extraction. Samples were stored at room temperature prior to analysis.

For flavonoid extraction, flavonoids were extracted using an ultrasonic-assisted method with minor modifications (Huang et al. 2009). Briefly, 50 mL of 75 % ethanol was added to each sample and the extraction was conducted for 45 min at 65 °C.

The total flavonoid concentration was determined by using a colorimetric method with minor modification (Bao et al. 2005). About 2.0 g sample was placed in a soxhlet extractor and refluxed with petroleum ether for 4 h at 80 °C to remove fat soluble impurities. The extract was discarded, and the retained residues were dried at room temperature. The extract was evaporated to dryness in a rotary vacuum evaporator at <40 °C and then dissolved with methanol. Exactly 0.3 mL of 5 % NaNO_2_ was added to a 1 mL extract in a 10 mL volumetric flask, and the mixture was kept for 5 min at room temperature. The residue was extracted two times and the obtained extract was combined and condensed to 10 mL. Addition of 0.3 mL of 10 % AlCl_3_·6H_2_O to the mixture, which was incubated for another 5 min, was followed by the addition of 2 mL of 1 M NaOH. After 15 min of incubation at room temperature for color development, the absorbance at 415 nm was measured. Total flavonoid concentration was calculated using the standard rutin curve and expressed as milligrams rutin equivalent per gram of dry mass (mg/g dm).

Analysis of selected flavonoids (kaempferol, quercetin and isoquercitrin) was performed using high-performance liquid chromatography. All obtained extracts were filtered through a 0.45 um polytetrafluoroethylene (PTFE) filter prior to HPLC analysis. An Agilent 1200 series HPLC system (Waldbronn, Germany), which consists of an online degasser, a quaternary pump solvent management system, an autosampler, a column heater, an UV/VIS diode array detector (DAD), and a data processing system, was used for the flavonoid separation. Quercetin and kaempferol were quantified as aglycones after acid hydrolysis. The hydrolyzed extracts were separated on an Eclipse Plus C18 column (250 mm × 4.6 mm, 5 um) at 30 °C. The mobile phases were methanol (A) and 0.3 % phosphoric acid (B) at 55: 45 (VA/VB). The flow rate was 1.0 mL min^−1^and the wavelength of detection was 365 nm. For isoquercitrin determination, the mobile phases were methanol (A) and 0.5 % phosphoric acid (B). The gradient elution included 0–25 min, 15 % A; 15–26 min, 15–90 % A; 26–36 min, 90 % A; 36–37 min, 90–15 % A; and 37–45 min, 25 % A. The detection wavelength was 350 nm. The column temperature and flow rate were similar to the conditions described for the detection of quercetin and kaempferol. The standards of quercetin, kaempferol (Sigma–AldrichInc., St. Louis, USA), and isoquercitrin (National Institute for the Control of Pharmaceutical and Biological Products, Beijing, China) were used to obtain an external calibration curve.

### Statistical analysis

Data are reported as the mean ± standard deviation (SD), and all tests were performed using the SPSS 16.0 statistical software program (SPSS, Chicago, IL, USA). A two-way ANOVA model with shading and provenance as the main fixed factors plus a shading × provenance interaction term, followed by Tukey’s multiple-range test, was performed for each leaf characteristic, flavonoid concentration, plant growth, and flavonoid accumulation per plant. Relationships be-tween leaf characteristics, individual environmental factors (solar radiation, air temperature, and RH values) and leaf flavonoid concentration were evaluated using the Pearson’s correlation analysis. The data were tested for normality (Shapiro–Wilk normality test) before analysis of variance. All statistical analyses were performed at a 95 % confidence level.

## Results

### Variation in environmental factors

The microclimatic parameters varied in different shading conditions. From June 20 to October 20, values of daily mean air temperature in the three shading treatments were 31.04 ± 4.92 °C for A1, 29.39 ± 4.99 °C for A2, and 27.41 ± 4.11 °C for A3, whereas the daily mean RH values were 63.2 ± 16.25, 69.82 ± 4.99, and 74 ± 16.08 % for A1, A2, and A3, respectively. Also, a great difference in the range of PPFD was observed at the same time. The distribution of PPFD in treatment A1 was 29.2 % in 0–200 μmol/m^2^/s, 25.3 % in 201–400 μmol/m^2^/s, 14.5 % in 401–600 μmol/m^2^/s, 10.2 % in 601–800 μmol/m^2^/s, 6.6 % in 801–1000 μmol/m^2^/s, and 14.2 % in >1000 μmol/m^2^/s during the 4 months. The PPFD in treatment A2 ranged from 0 to 600 μmol/m^2^/s, and the distribution was 73.3 % in 0–200 μmol/m^2^/s, 20.3 % in 201–400 μmol/m^2^/s, and 5.4 % in 401–600 μmol/m^2^/s, whereas the greatest PPFD distribution in treatment A3 was in 0–200 μmol/m^2^/s (accounting for 98.2 %) in the same periods.

### Variation in leaf characteristics

**Fig. 1:**
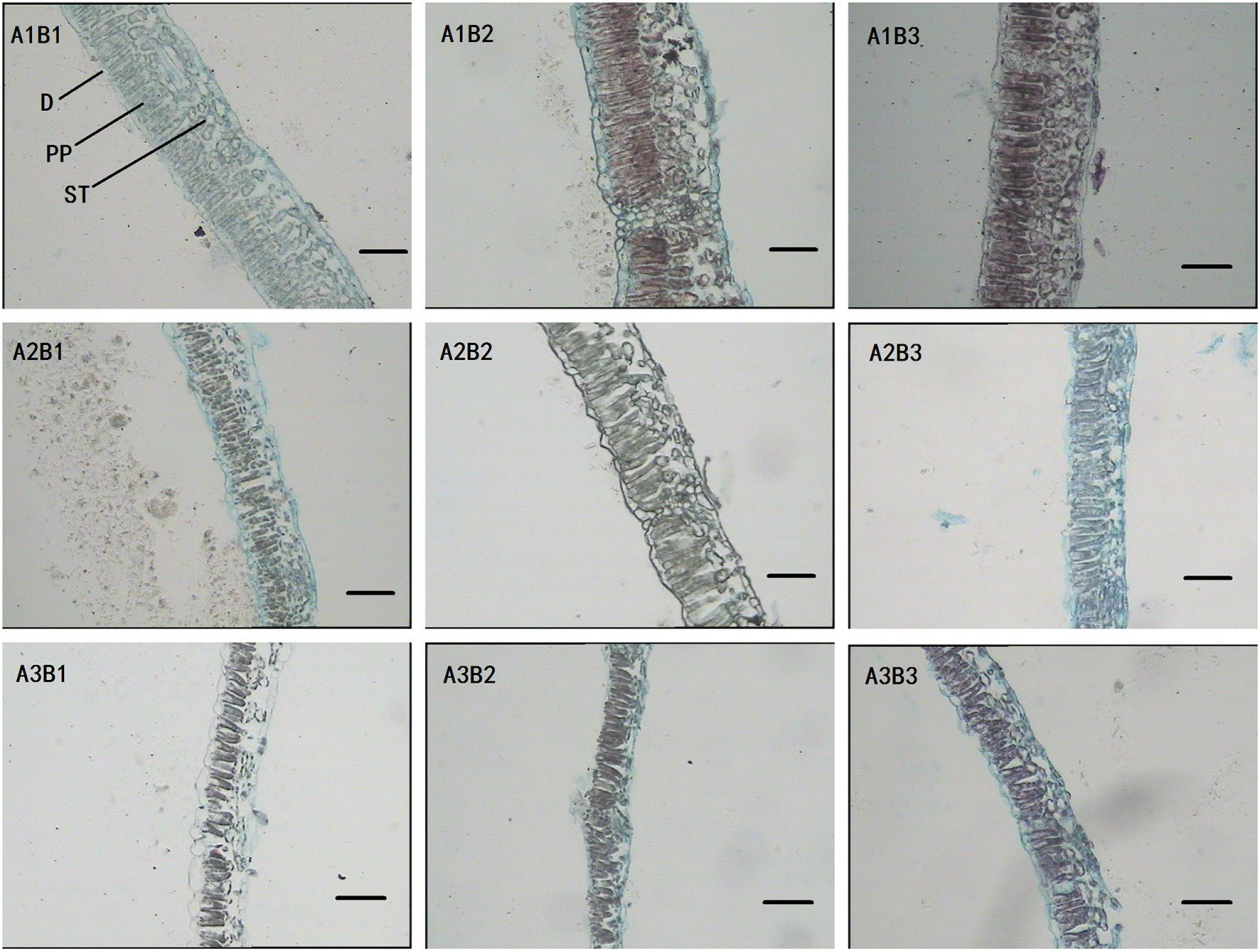
Anatomical structure of *Cyclocarya paliurus* leaves under different treatments (showed for cross sections. D, PP and ST indicate adaxial epidermis, palisade parenchyma and spongy tissue, respectively). *Scale* 100 μm

Leaf morphology and growth of *C. paliurus* were found to be significantly different under various provenance and shading treatments (Table 1). Moreover, a significant interaction of light intensity and provenance was observed in palisade length and ratio of chlorophyll a to b (Chla/b ratio) of *C. paliurus* (Table 2). One layer shading resulted in the highest LA, and LA was significantly higher in B3 treatment (Muchuan provenance) than in the other two provenances for all light conditions (Table 3). In the nine treatments, the highest LA value was observed in treatment A2B3 (1610 cm^2^), whereas the lowest value was detected in treatment A1B2 (793 cm^2^). Leaf thickness and length of palisade cells were significantly decreased under shading conditions, compared with full light treatment (Table 1). Compared with other provenances, B2 treatment (Yuanling provenance) under full light had the longest length of palisade cells (Fig. 1; Table 3). There were higher total pigments concentrations and smaller Chla/b ratio in shaded plants than in plants grown under full sunlight (Table 1). In term of provenance, B2 treatment (Yuanling provenance) presented the lowest pigments concentrations and the smallest Chla/b ratio.

**Table 1:** Leaf characteristics and chlorophyll concentration of *Cyclocarya paliurus* leaves under different treatments

Treatment	Leaf area per plant (cm^2^)	Leaf thickness (μm)	Palisade length (μm)	Total chlorophyll concentration (mg g^−1^)	Chlorophyll ratio *a/b*
A1B1	846 ± 25.8a	244.1 ± 19.5d	110.1 ± 4.5c	1.69 ± 0.28abc	4.00 ± 0.02e
A1B2	793 ± 15.8a	301.0 ± 20.6e	134.7 ± 2.2d	1.43 ± 0.16a	3.54 ± 0.04c
A1B3	1158 ± 23.2abc	257.2 ± 9.1d	123.1 ± 8.2cd	1.35 ± 0.24a	3.96 ± 0.20e
A2B1	1299 ± 177.3abc	135.5 ± 24.4ab	79.0 ± 6.3b	2.19 ± 0.22d	3.78 ± 0.06d
A2B2	867 ± 79.5a	170.2 ± 1.5c	78.2 ± 4.3b	1.63 ± 0.08ab	3.43 ± 0.03bc
A2B3	1610 ± 115.6c	126.0 ± 14.2ab	60.8 ± 3.2a	1.94 ± 0.29bcd	3.56 ± 0.06c
A3B1	959 ± 16.0ab	105.0 ± 19.7a	55.8 ± 6.3a	2.15 ± 0.21cd	3.55 ± 0.03c
A3B2	1156 ± 48.1abc	147.8 ± 17.2ab	76.1 ± 7.5b	1.79 ± 0.24abcd	3.25 ± 0.04a
A3B3	1384 ± 43.3bc	116.3 ± 1.1a	50.0 ± 6.5a	2.08 ± 0.11cd	3.38 ± 0.06b

Mean ± SD in the same column with different letters are statistically significantly different among the treatments for each determined index (*P* < 0.05 by Tukey’s test)

**Table 2:** Summary of significance levels (Two-way ANOVA) for the effects of shading, provenance and their interaction on leaf characteristics and chlorophyll concentration of *Cyclocarya paliurus* leaves

Source	Leaf area (cm^2^)	Leaf thickness (μm)	Palisade length (μm)	Total chlorophyll concentration (mg g^−1^)	Chlorophyll *a/b*
Shading (A)					
f	2	2	2	2	2
MS	5269.2	55,460.7	9643.7	0.6945	0.4441
P	*0.049*	*<0.001*	*<0.001*	*<0.001*	*<0.001*
Provenance (B)					
f	2	2	2	2	2
MS	67,430.7	3103.7	447.9	0.0159	0.1599
P	*0.006*	*<0.001*	*0.001*	*0.012*	*<0.001*
Interaction (A × B)					
f	4	4	4	4	4
MS	743.1	38.2	59.8	0.0025	0.0125
P	0.331	0.647	*0.040*	0.561	*0.029*

Italic values indicate the treatment effects are statistically significant at the 0.05 level for each determined index

**Table 3:** Tukey’s multiple-range test of leaf characteristics and chlorophyll concentration of *Cyclocarya paliurus* leaves after a Two-way ANOVA

Treatment	Level	Leaf area (cm^2^)	Leaf thickness (μm)	Palisade length (μm)	Total chlorophyll concentration (mg g^−1^)	Chlorophyll *a/b*
Shading (A)	A1	932a	267.4c	122.7c	1.49a	3.83c
	A2	1259b	143.9b	72.7b	1.92b	3.59b
	A3	1167ab	123.0a	60.6a	2.01b	3.39a
Provenance (B)	B1	1035a	161.6a	81.6a	2.01c	3.78c
	B2	939a	206.3b	96.3b	1.62a	3.40a
	B3	1384b	166.5a	78.0a	1.79ab	3.63b

### Variation in leaf flavonoid concentration and seasonal dynamics

Two-way ANOVA showed that shading significantly affected concentrations of total and selected flavonoids in *C. paliurus* leaves, while the concentrations of quercetin and isoquercitrin were significantly affected by provenance and the interaction between shading and provenance (Table 4). The highest concentrations of both total flavonoid and kaempferol concentrations were observed in treatment A1B3, while the highest concentrations of quercetin and isoquercitrin were found in treatment A1B2. However, no significant difference in concentrations of total flavonoid, isoquercitrin and kaempferol was found under shading conditions (Table 5). Compared with full light treatment, mean concentrations of total flavonoid, quercetin, kaempferol, and isoquercitrin in *C. paliurus* under one layer shading significantly decreased by 41.1, 74.1, 67.0 and 80.2 %, respectively. On the levels of provenance, B2 treatment (Yuanling provenance) under full light condition achieved the highest concentrations of quercetin and isoquercitrin, while B3 treatment (Muchuan provenance) under full light condition obtained the highest concentrations of total flavonoid and kaempferol.

**Table 4:** Summary of significance levels (Two-way ANOVA) for the effects of shading, provenance and their interaction on biomass accumulation, flavonoid concentrations and flavonoid yield in leaves of *Cyclocarya paliurus*

Source	Biomass accumulation (g plant^−1^ dm)				Flavonoid concentrations (mg g^−1^ dm)				Flavonoid yield (mg plant^−1^ dm)			
	Root	Stem	Leaf	Total	Total	Quercetin	Isoquercitrin	Kaempferol	Total	Quercetin	Isoquercitrin	Kaempferol
Shading (A)												
f	2	2	2	2	2	2	2	2	2	2	2	2
MS	7332.3	10,952.4	2977.3	58,209.7	806.7	9.4058	24.425	4.979	6,077,330.0	28,472.3	59,931.2	9511.8
P	*<0.001*	*<0.001*	*<0.001*	*<0.001*	*<0.001*	*<0.001*	*<0.001*	*<0.001*	*<0.001*	*<0.001*	*<0.001*	*<0.001*
Provenance (B)												
f	2	2	2	2	2	2	2	2	2	2	2	2
MS	1827.4	4905.5	2986.8	35,337.9	19.2	2.3303	2.244	0.0197	3,853,934.2	16,248.5	19,977.7	2298.6
P	*<0.001*	*<0.001*	*<0.001*	*<0.001*	0.234	*<0.001*	*<0.001*	0.130	*<0.001*	*<0.001*	*<0.001*	*<0.001*
Interaction												
f	4	4	4	4	4	4	4	4	4	4	4	4
MS	1635.3	4912.3	2940.5	27,709.1	4.9	0.1994	1.194	0.1436	4,847,762.0	7018.2	25,089.3	1907.3
P	*0.008*	*0.001*	*0.005*	*<0.001*	0.970	*0.002*	*0.001*	0.078	*<0.001*	*0.004*	*<0.001*	*<0.001*

Italic values indicate the treatment effects are statistically significant at the 0.05 level for each determined index

**Table 5:** Flavonoid concentrations in leaves of *Cyclocarya paliurus* under various treatments of shading and provenance (mean ± SD, samples collected in October 20, 2011)

Treatment	Flavonoid concentrations (mg g^−1^)			
	Total	Quercetin	Isoquercitrin	Kaempferol
A1B1	40.9 ± 4.6b	2.11 ± 0.19c	2.37 ± 0.32b	2.12 ± 0.79b
A1B2	42.6 ± 2.8b	2.13 ± 0.03c	4.35 ± 0.53d	2.10 ± 0.01b
A1B3	45.8 ± 9.3b	1.28 ± 0.31b	3.67 ± 0.64c	2.97 ± 0.35c
A2B1	24.8 ± 3.4a	0.62 ± 0.05a	0.53 ± 0.07a	0.87 ± 0.08a
A2B2	23.3 ± 2.2a	0.40 ± 0.04a	0.76 ± 0.07a	0.59 ± 0.06a
A2B3	28.0 ± 6.1a	0.41 ± 0.08a	0.77 ± 0.20a	0.91 ± 0.17a
A3B1	27.6 ± 1.8a	0.54 ± 0.05a	0.49 ± 0.06a	0.71 ± 0.07a
A3B2	28.8 ± 6.4a	0.50 ± 0.13a	1.06 ± 0.16a	0.44 ± 0.01a
A3B3	30.7 ± 0.6a	0.25 ± 0.03a	0.50 ± 0.05a	0.35 ± 0.03a

Different letters indicate significant differences among treatments for the same category according to Tukey’s test (*p* < 0.05)

**Fig. 2:**
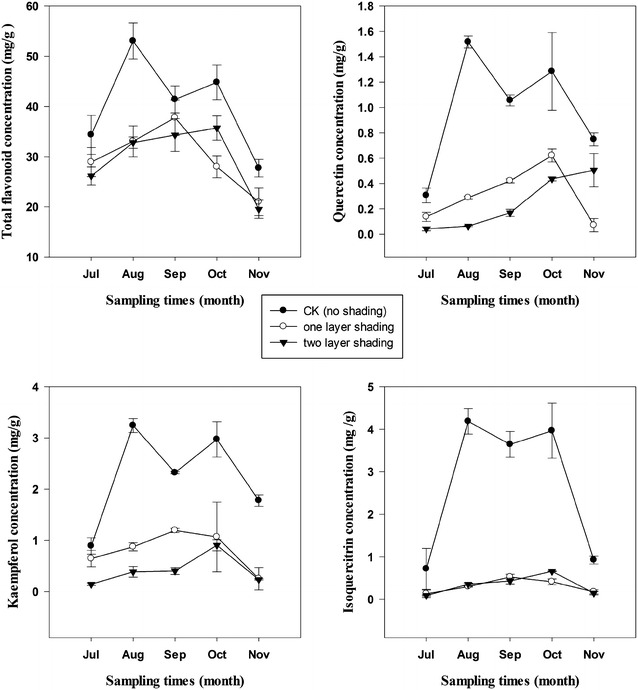
Seasonal variation in concentrations of total and selected flavonoids (quercetin, kaempferol, and isoquercitrin) in leaves of *Cyclocarya paliurus* under three shading treatments (based on the means of three provenances) (mean ± SD)

Based on the means of three provenances, seasonal variation in total flavonoid concentration of full light treatment showed a bimodal temporal pattern with peaks of 53.04 mg g^−1^ in August and 44.81 mg g^−1^ in October, which were 91.2 and 61.5 % higher than the concentration in November respectively when the lowest flavonoid concentration (27.74 mg g^−1^) occurred (Fig. 2). This trend persisted across the key health promoting flavonoids (quercetin, kaempferol, and isoquercitrin). Shading treatment obviously changed the seasonal dynamics of leaf flavonoid concentrations (Fig. 2). The highest concentrations of total flavonoid, isoquercitrin and kaempferol under A2 and A3 treatments were observed in September and October respectively, while the highest concentration of quercetin under A2 and A3 treatments occurred in October and November.

### Variation in biomass production and allocation

Biomass production of *C. paliurus* coppices was significant affected by shading, provenance and their interaction (Table 4; p < 0.05). The total biomass production per plant among the integrated treatments was in the order A1B3 > A2B3 > A1B1 > A1B2 > A2B2 > A2B1 > A3B2 > A3B3 > A3B1, and this trend persisted across the growth of leaf, stem, and root (Table 6). Across the nine treatments, biomass allocation for root, stem, and leaf components was 36.0, 41.8 and 23.2 %, respectively. However, there existed some differences in biomass allocation among the nine treatments. The highest ratio of root to total biomass was observed in treatment A3B2 (42.8 %), whereas the greatest ratio of shoot to total biomass was achieved in treatment A2B3 (74.8 %). Compared to the mean value of nine treatments, leaf production per plant in A3B1, A3B3, A3B2, A2B1, A1B1, A1B2 and A2B2 treatments decreased by 80.5, 69.5, 64.0, 59.9, 25.0, 24.0 and 10.4 %, whereas in A2B3 and A1B3 treatments increased by 147.1 and 186.5 %, respectively.

**Table 6:** Variation in biomass production of *Cyclocarya paliurus* under different treatments of shading and provenance (mean ± SD, samples collected in October 20, 2011)

Treatment	Biomass production (g)			
	Root	Stem	Leaf	Total
A1B1	57.9 ± 4.00cd	62.9 ± 7.08cd	21.6 ± 2.72a	142.5 ± 13.8bc
A1B2	47.8 ± 5.42cd	46.4 ± 4.70bc	21.9 ± 2.48a	112.6 ± 12.6b
A1B3	104.5 ± 9.50d	129.3 ± 13.76d	82.7 ± 8.75b	316.5 ± 32.02d
A2B1	20.1 ± 2.08bc	30.8 ± 2.36b	11.5 ± 1.73a	62.5 ± 6.18ab
A2B2	36.9 ± 3.22bcd	40.4 ± 3.25bc	25.8 ± 2.05a	103.1 ± 8.53ab
A2B3	59.8 ± 5.50 cd	106.0 ± 10.27d	71.3 ± 6.67b	237.2 ± 22.46cd
A3B1	9.4 ± 1.48a	11.3 ± 0.82a	5.6 ± 0.47a	26.4 ± 2.77a
A3B2	17.2 ± 1.92b	12.6 ± 0.77a	10.3 ± 0.74a	40.3 ± 3.44ab
A3B3	12.6 ± 2.18b	10.7 ± 0.98a	8.8 ± 0.46a	32.2 ± 2.59a

Different letters indicate significant differences among treatments for the same category according to Tukey’s test (*p* < 0.05)

### Variation in flavonoid accumulation

**Fig. 3:**
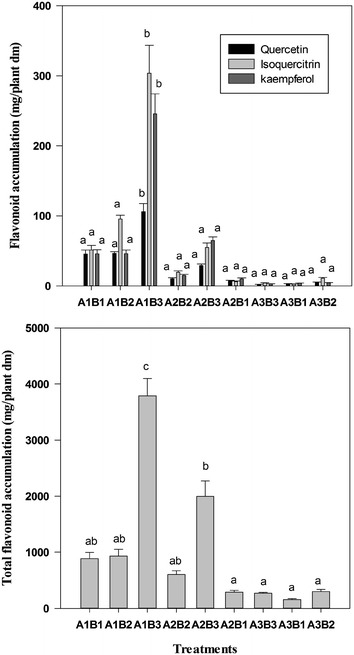
Variation in total and selected flavonoid accumulation in leaves of *Cyclocarya paliurus* per plant among various treatments (mean ± SD, samples collected in October 20, 2011). Different lower case letters indicate significant differences among various treatments within the same flavonoid (p < 0.05 by Tukey’s test). A1, A2 and A3 represent no shading, shading with one-layer net, and shading with two-layer nets, respectively, whereas B1, B2 and B3 indicate different provenances Wufeng, Yuanling and Muchuan, respectively

Based on the leaf biomass and flavonoid concentration, total and selected flavonoid accumulations in the leaves per plant were calculated for nine treatments. Two-way ANOVA indicated that an integrated treatment effect on the accumulations of both total and selected flavonoids in leaves per plant was significant (p < 0.05; Fig. 3). The greatest accumulations of total and selected flavonoids in the leaves per plant were achieved in treatment A1B3, followed by treatment A2B3, whereas the lowest were found in treatments A3B1 and A3B3. Compared to treatment A1B3, the total flavonoid accumulation in other treatments was decreased by 47.9–95.9 %, whereas the accumulations of kaempferol, quercetin, and isoquercitrin were decreased by 73.6–98.7, 55.9–97.9, 68.6–98.1 % in other treatments, respectively. Moreover, a two-way ANOVA showed that light intensity and provenance as well as their interaction significantly affected the accumulations of total and selected flavonoids in *C. paliurus* (Table 4).

## Discussion

### Effects of light regime and provenance on leaf characteristics

Photosynthetic light acclimation involves a variety of responses, including changes in leaf anatomy (Weston et al. 2000). The palisade parenchyma and spongy parenchyma in the leaf mesophyll are important photosynthetic tissues. Palisade tissue enables a better light penetration to the chloroplasts, while spongy tissue enhances the light capture by scattering light (Evans 1989). In the current study, higher light intensity seemed to positively affect the leaf growth and structure of the palisade parenchyma of *C. paliurus,* as the maximum length of palisade cells was observed under full light conditions (Table 1; Fig. 1). This indicated that the adjustment of palisade lengths under different light conditions is part of photosynthetic acclimation. Plants under full light conditions had a significantly higher biomass accumulation, while insufficient light under shading treatments resulted in reduced net carbon gain and plant growth (Table 6). Larger leaves and increased pigments concentrations in shading treatments may be a response to low photosynthetic photon flux density (PPFD), as larger leaves and more pigments would improve light interception and absorption for photosynthesis when light levels are low (Delagrange et al. 2006; Peri et al. 2007; Valladares and Niinemets 2008). In addition, the reduction in the Chla/b ratio was observed under shade treatments mainly due to the increase in Chlb concentration (Tables 1, 3), which contributes to the light capture for photosynthesis (Murchie and Horton 1997). These findings are similar to the results reported in other species (Zhao et al. 2012; Ma et al. 2015; Tang et al. 2015).

A two-way ANOVA showed that both light intensity and provenance significantly affected the morphology and leaf growth index of *C. paliurus* (Table 2). However, no significant interaction of light intensity and provenance was observed in LA, leaf thickness and total chlorophyll concentration of *C. paliurus*. The results from this study also showed that *C. paliurus* of Muchuan provenance presented a better adjustment to shading conditions compared with Wufeng provenance and Yuanling provenance, as the largest value of LA was observed in shading treatments of Wufeng provenance (Tables 1, 3), which indicated that provenance selection is important for planting *C. paliurus* under different environments.

### Effects of light regime and provenance on growth and flavonoid concentration

It is generally recognized that light intensity plays an important role in plant growth and photosynthetic capacity (Gottschalk 1994). Insufficient light may stress plant growth by limiting photosynthesis, while high light levels may damage the photosynthetic apparatus (Tang et al. 2015). The present study demonstrated that shading had significant negative effects on leaf and total biomass production of *C. paliurus* coppices, while full light condition resulted in the highest growth for all the components, indicating that *C. paliurus* is a heliophyte. The observed growth response of *C. paliurus* to light intensity was similar to that of many tree species growing under various light intensities (Poorter 1999; Cai et al. 2009; Ma et al. 2015; Tang et al. 2015). The present study also indicated that leaf biomass ratio of *C. paliurus* increased at lower light intensity, whereas full sunlight increased the root and stem biomass ratios, respectively (Table 6). The reason might be that higher transpiration rate leads to increasing root biomass allocation in order to improve water uptake ability under full sunlight conditions, in agreement with that reported on *Rauvolfia* species, *Anoectochilus formosanus* Hayata, and freshwater macrophytes (Cai et al. 2009; Ma et al. 2010; Cronin and Lodge 2003).

Light intensity is known to affect not only plant growth and development but also the biosynthesis of both primary and secondary metabolites (Hemm et al. 2004). Fang et al. (2011) reported that genotypes play a significant role in flavonoid accumulation of *C. paliurus* grown under the same environment. In the present study, a two-way ANOVA showed that shading significantly affected concentrations of total and selected flavonoids in *C. paliurus* leaves, and meanwhile provenance and the interaction between shading and provenance also significantly affected the concentrations of quercetin and isoquercitrin. However, provenance and the interaction had no significant effects on concentrations of total flavonoid and kaempferol (Table 4). Differences in flavonoid concentration were significant among the five sampling times, showing that flavonoid accumulation was strongly affected by seasonal progression. This finding suggests that shading treatments not only reduced the leaf concentrations of flavonoids but also delayed the appearance of the highest flavonoid concentrations in *C. paliurus* leaf of different provenances during the growing season. Seasonal changes of flavonoid concentrations under different light conditions also indicated that the contents of flavonoids were highly positively correlated with total solar radiation in the period from planting to the sampling time, in agreement with the results from previous study (Gliszczyńska-Świgło et al. 2007). Overall, shading had a significantly negative effect on the accumulation of total flavonoids and the studied flavonoids (Table 5), supporting that high photosynthetic active radiation (PAR) triggers biosynthesis of flavonoids (Xu et al. 2014).

Accumulation of phytochemical compounds in plants is often induced by environmental factors. For example, visible light primarily induces biosynthesis of proanthocyanidins and affects their composition, whereas UV light specifically induces biosynthesis of flavonols (Koyama et al. 2012). Compared with genetic effects, light intensity seemed to have greater influence on flavonoid biosynthesis (Table 4). Björkman et al. (2011) and Edreva (2005) reported that flavonoids in plants may carry defensive functions against environmental stresses, especially higher light radiation, which is evidenced by data obtained in full light treatments of our study. Moreover, strong correlations were found between monthly light intensity and measured flavonoid concentrations in the present study (Table 7). In addition, significantly positive correlations were found between leaf thickness, palisade length and flavonoid concentrations (Table 8). To some extent, these results reflected that light conditions influenced both leaf growth and accumulation of phytochemical compounds in *C. paliurus*. In the present study, Muchuan provenance (B3) which origined from lower latitude and higher altitude achieved the highest flavonoid contents. This indicated that both environmental factors and genotype have great contribution to the accumulation of phytochemical compounds, as reported in other plants (Adom et al. 2003; André et al. 2009; Schmidt et al. 2010). Thus, it could be expected that manipulation of light intensity and genotypes would be a powerful tool for stimulating secondary plant metabolite accumulation, particularly for crops in intensive management system.

**Table 7:** Pearson correlation coefficients between environmental parameters and leaf flavonoid concentrations (n = 27)

Environmental factor	Flavonoid concentrations (mg g^−1^)			
	Total	Quercetin	Isoquercitrin	Kaempferol
Monthly light intensity (μmol m^−2^ s^−1^)	0.580*	0.771**	0.780**	0.785**
Monthly air temperature (°C)	0.240	0.298	0.481	0.292
Monthly relative humidity (%)	−0.257	−0.404	−0.316	−0.439

* and ** indicate correlation is significant at the 0.05 and 0.01 level, respectively

**Table 8:** Pearson correlation coefficients between leaf characteristics and leaf flavonoid concentrations (n = 27)

Leaf characteristics	Flavonoid concentrations (mg g^−1^)			
	Total	Quercetin	Isoquercitrin	Kaempferol
Leaf area per plant (cm^2^)	−0.334	−0.614	−0.469	−0.339
Leaf thickness (μm)	0.859**	0.904**	0.962**	0.866**
Palisade length (μm)	0.829**	0.887**	0.948**	0.884**
Total chlorophyll concentration (mg g^−1^)	−0.729*	−0.636	−0.853**	−0.746*
Chlorophyll ratio a/b	0.574	0.591	0.443	0.777*

* and ** indicate correlation is significant at the 0.05 and 0.01 level, respectively

### Effects of light regime and provenance on flavonoid production

Plant growth and content of phytochemicals are affected by genetic, management practice, harvesting and environment factors that occur during the growing period (Graham 1998; Björkman et al. 2011; Deng et al. 2012). Two-way analysis of variance indicated that biomass accumulation of *C. paliurus* coppices was not only significantly influenced by light intensity, but also significantly by genotype and interactions between these two variables (Table 4). The present study indicated that B3 treatment (Muchuan provenance) achieved the highest total biomass, and the highest leaf biomass in all shading conditions, compared with other provenances. Thus, provenance selection is crucial to optimize higher flavonoids yield of *C. paliurus* coppices, as leaf biomass has a great contribution to the accumulation of total flavonoid and key health promoting flavonoids (quercetin, kaempferol, and isoquercitrin) per plant.

In leaf-harvest plantations of *C. paliurus* coppice, the goal is to obtain not only higher quality (higher flavonoid concentrations) but also higher yield (equal to flavonoid concentrations multiplied by leaf biomass), thus both provenance selection and the optimal silvicultral measures are important for production practices aimed at obtaining higher flavonoids yields (Poutaraud and Girardin 2005). However, the response may be quite different and also vary for different crops and secondary metabolites. For example, fertilizer application did not promote polyphenolic formation in apples (Morales-Sillero et al. 2008) and leaf flavonoid content in *C. paliurus* (Deng et al. 2012), whereas leaf phenolics concentrations were 31 % higher in fertilized than in unfertilized plants of freshwater macrophytes (Cronin and Lodge 2003). In the present study, the A1B3 treatment (full light and Muchuan provenance) was the most effective way to improve the accumulations of total and selected flavonoids because it resulted in the highest leaf biomass production. Overall, in order to achieve the highest flavonoid yield per area for food and medicinal utilization, it is important to manipulate field-growing conditions such as light intensity and select genotypes in *C. paliurus* coppices. However, more provenance trials are required to clarify the genotype effects of this species at different sites.

In conclusion, significant variations in leaf morphology, growth and accumulations of total and selected flavonoids were found in *C. paliurus* growing under light intensity and genotype treatments. Both light regime and provenance significantly affected the morphology and growth index of *C. paliurus* leaves. In the full light condition, leaf flavonoid concentrations showed a bimodal variation pattern. However, shading treatments not only reduced the leaf concentrations of flavonoids but also delayed the peak appearing of the flavonoid concentrations in *C. paliurus* leaf of different provenances. Muchuan provenance with full light achieved the highest leaf biomass and flavonoid accumulation. Moreover, documentation of genotypic variation among different light environments provides a basis for extensive selection and breeding programs in improving the leaf concentrations of flavonoids compounds of *C. paliurus*.
